# Ethnicity and risk of diagnosed dementia after stroke: a cohort study using the Clinical Practice Research Datalink

**DOI:** 10.1136/jech-2019-212825

**Published:** 2019-11-07

**Authors:** Suhail Ismail Shiekh, Harriet Forbes, Rohini Mathur, Liam Smeeth, Neil Pearce, Charlotte Warren-Gash

**Affiliations:** 1 Department of Non-Communicable Disease Epidemiology, London School of Hygiene and Tropical Medicine Faculty of Epidemiology and Population Health, London, UK; 2 Department of Medical Statistics, London School of Hygiene and Tropical Medicine Faculty of Epidemiology and Population Health, London, UK

**Keywords:** stroke, dementia, ethnicity, epidemiology

## Abstract

**Introduction:**

The UK has over 1.2 million stroke survivors. Stroke is a major risk factor for dementia, and along with other risk factors such as hypertension and diabetes, is more common among Black, Asian and other ethnic minorities (BAME). We aimed to explore whether diagnosed dementia differed by ethnicity among adult stroke survivors.

**Methodology:**

Using the UK Clinical Practice Research Datalink and linked hospital data, we conducted a cohort study among patients aged ≥40 years who had an incident stroke between 2005 and 2016. We fitted multivariable Cox proportional hazard models to estimate ethnic differences in the risk of poststroke dementia, adjusting for major clinical and social confounders.

**Results:**

Our cohort comprised 45 474 stroke survivors (mean age 72.6 years, 49% female), of whom 95.7% were White, 2.0% South Asian, 1.2% Black and 1.1% of Mixed/Other ethnicity. Of these, 4624 (10.2%) were diagnosed with poststroke dementia over a median follow-up of 3.26 years. Compared with the White ethnic group, those of Black ethnicity were 42% more likely to be diagnosed with dementia (adjusted HR 1.42, 95% CI 1.05 to 1.93). There was no association between any other ethnic group and poststroke dementia diagnosis.

**Discussion:**

There was good evidence that those of Black ethnicity had higher risk of diagnosed dementia poststroke. Further understanding of the mechanisms of this relationship could help target interventions at communities most at risk of dementia poststroke.

## Introduction

The UK has over 1.2 million stroke survivors,^1^ about two-thirds of whom are affected by disabilities,^1^ including cognitive impairments. Improving cognition has been identified as the most important issue for stroke survivors, caregivers and health professionals in a recent national priority setting exercise using James Lind Alliance methodology.^2^

About one-third of stroke survivors develop dementia.^3^ Stroke is a major risk factor for dementia and both stroke incidence and mortality are markedly higher among South Asian and Black populations compared with those from White ethnic groups.^4^ This might be partly explained by differences in stroke risk factors such as diabetes, hypertension and smoking patterns, but is also likely to reflect complex social, economic and behavioural differences.^5^ In addition, dementia is thought to be more common in people from Black, Asian and other ethnic minorities (BAME).^6^ According to data from the USA, the Black ethnic group has a higher risk of developing dementia than the general population.^7^ Some small cross-sectional studies from the UK also indicate higher prevalence of dementia in the African-Caribbean ethnic group compared with the White ethnic group.^8 9^ One further study has shown that people of Asian ethnicity in the UK are less likely to be diagnosed with dementia than those of White ethnicity.^10^

However, because previous studies have focused on all-cause dementia it is unclear whether there are ethnic differences in the risk of poststroke dementia (defined as any dementia occurring after stroke, with common causes including vascular lesions, Alzheimer’s pathology and white matter changes),^11^ which might be more amenable to prevention than other types of dementia. While there is some evidence that poststroke dementia is higher in BAME communities,^6^ this subject is under-researched and has not been studied in a UK setting. The proportion of elderly people from BAME is set to increase in the future as the UK population ages. The number of people from BAME with dementia is projected to increase sevenfold by 2051 compared with a twofold increase in the general population^12^ due to population growth and ageing.

It is therefore important to understand ethnic differences in poststroke dementia risk to help inform service provision and research into preventative interventions for these groups. We aimed to explore whether dementia diagnosis in adult stroke survivors differed by ethnicity in the UK and we hypothesised that the incidence of diagnosed poststroke dementia would be higher in BAME communities.

## Methods

### Data source

We used data from the Clinical Practice Research Datalink (CPRD) (January 2017 build) linked to Hospital Episode Statistics (HES) records of admitted patient care. The CPRD includes read-coded electronic health record data from 670 general practices in the UK on diagnoses, investigations and referrals in addition to patient demographic data. The CPRD covers about 8% of the UK population and is broadly representative with respect to age, gender, ethnicity and mortality rates. HES comprises ICD-10 coded records of care from NHS healthcare providers in England. About 75% of English CPRD practices have HES linked data.

### Study design and population

We conducted a cohort study using data available from people who had survived a first incident stroke as recorded in CPRD or HES between 1 January 2005 and 31 December 2016, occurring at the age of 40 years or above, after at least 12 months of research-standard CPRD follow-up. We excluded those with a history of dementia prior to or at the time of stroke, or dementia occurring within 3 months of stroke, along with those who died or transferred out of the practice in the 3-months poststroke. Our study population included only patients who had a stroke. We used a broad definition for stroke including ICD-10 codes for subarachnoid haemorrhage, intracerebral haemorrhage, cerebral infarction and stroke not specified as haemorrhage or infarction. We identified stroke patients in HES data using these codes and in CPRD using read codes (online supplementary file code lists 3–4).

10.1136/jech-2019-212825.supp1 Supplementary data

### Definition of exposure: ethnicity

We developed a nominal categorical variable using a previously developed algorithm^13^ to define ethnicity status in CPRD and HES. Where ethnicity data was missing in CPRD data it was updated using HES data where available. We categorised ethnicity into White, South Asian, Black African/Caribbean and Mixed/Other.

### Definition of outcome: dementia

Dementia diagnosed during the study period was the outcome of interest. We used a broad definition for dementia including ICD-10 codes for vascular dementia, Alzheimer’s disease, unspecified dementia and other degenerative diseases of the nervous system. We identified diagnosed dementia in HES data using these codes and in CPRD using read codes mapped to these ICD-10 codes (online supplementary file code lists 1–2). We did not include patients with prevalent codes for dementia events. We further classified diagnosed dementia into early dementia (3-months poststroke to 1 year), late dementia (from 1-year poststroke to 5 years) and very late dementia (>5-years poststroke).

### Covariates

Based on previous literature, we also included age (in years), sex (male or female), body mass index (BMI) (calculated from height and weight if available, or as entered directly), practice level Index of Multiple Deprivation (IMD) quintiles (quintile 1 was least deprived), smoking status (current smoker or non-current smoker (including former smokers)), alcohol consumption (non-drinker, current-drinker, ex-drinker). Comorbidities recorded at any time prior to stroke included: uncontrolled diabetes (binary, haemoglobin A1c (HbA1c) >7.5%), atrial fibrillation (binary, using codes), myocardial infarction (binary) and uncontrolled hypertension (binary, defined as systolic≥140 mm Hg and diastolic≥90 mm Hg). Other covariates included statin use within 2 years prior to stroke (binary), antiplatelet agent used poststroke as secondary prevention (binary), a prescription for immunosuppressive medication within 2 years prior to stroke (binary) and consultations per year in the 3-years prestroke.

### Follow-up

Patients were followed from 3 months after the date of stroke until the earliest of: dementia diagnosis; death; moving away from the general practice; last data collection from the practice; or the end of study period (31 December 2016). The first 3 months after stroke were excluded to avoid misclassification of reversible cognitive changes in this early period as dementia.

### Statistical analysis

We examined the distribution of the covariates by exposure (ethnicity) and the outcome (diagnosed dementia). We also fitted univariable Cox proportional hazard models with diagnosed dementia as the outcome with each exposure to examine crude associations.

We fitted multivariable Cox proportional hazard models to estimate HRs for dementia in patients of different non-White ethnicities taking the White ethnic group as the reference category and using age as the time scale. We examined the association between ethnicity and stroke after partial adjustment (for age as the time scale, sex and IMD) between ethnicity and poststroke dementia, and again after adjustment for all covariates in the full model (age as the time scale, sex, IMD, smoking status, alcohol consumption, atrial fibrillation, history of previous myocardial infarction, immunosuppressive prescription, statin use within 2 years prior to stroke, antiplatelet agent used post stroke as secondary prevention, and consultations per year in the 3-year prestroke). After the full model, we additionally adjusted for two potential mediators, uncontrolled hypertension and diabetes to explore effect of adjusting for these.

Dementia patients are commonly seen to be underweight possibly due to altered eating behaviours such as loss of appetite, forgetting or refusing to eat.^14^ Hence, we did not include BMI in our models in multivariable models as being underweight at the time of stroke was likely to be a proxy for the outcome, dementia. We assessed the strength of evidence for each association using p values from likelihood ratio tests. We also ran these analyses for early, late and very late dementia. Proportional hazards assumption was met for ethnicity using a statistical test in STATA based on Schoenfeld residuals.

We explored ethnic differences in premature exit from the study due to different factors (death within 3 months, transfer out not due to death within 3 months, dementia diagnosis within 3 months, transfer out due to all reasons) by modelling for these reasons as the outcomes.

We also conducted a sensitivity analysis by repeating the main analysis after restricting the population to only those with HES linked data in the follow-up period to compare to results from the primary analysis and identify changes in estimates, if any.

All analyses were conducted using STATA MP V.15.

## Results

### Description of cohort

**Figure 1:**
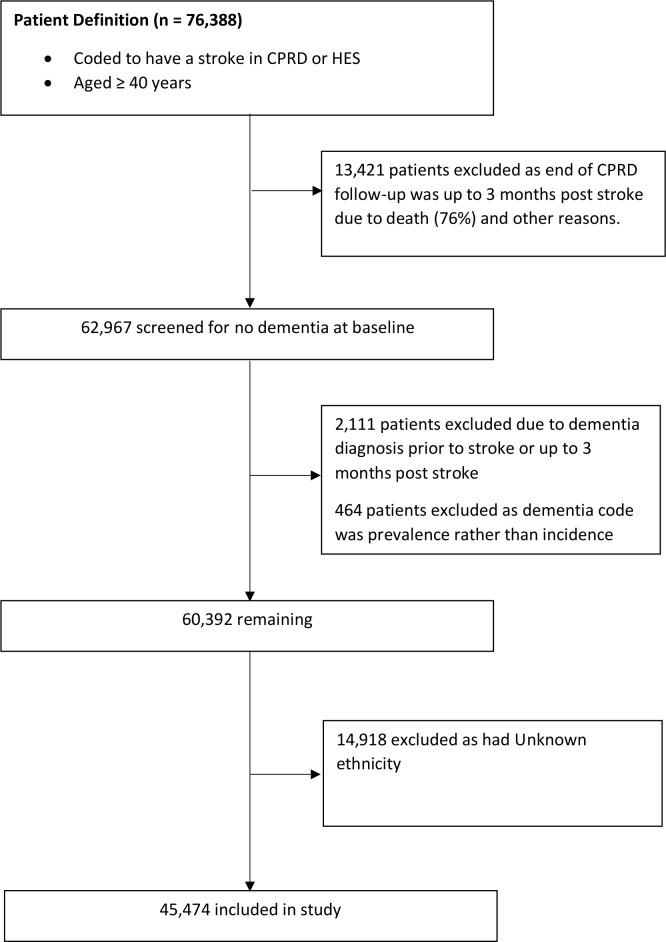
Flowchart of study population.

Figure 1 shows the steps followed in obtaining the sample of patients included in the study. Out of 76 388 patients with incident stroke, 15 996 patients were excluded as they had either transferred out of the practice due to death or other reasons within 3 months after stroke, had a history of dementia up to 3-months poststroke or had a prevalent rather than incident code for dementia.

Of the remaining 60 392, we had ethnicity information for 45 474 patients (75%) (mean age-at-stroke 72.6 years, 49% female), of whom 43 526 (95.7%) were White, 885 (2.0%) South Asian, 543 (1.2%) Black and 520 (1.1%) were of Mixed/Other ethnicities (table 1). The median follow-up was 3.26 years (IQR 0.96–4.98).

**Table 1:** Characteristics of poststroke study population

	N (%)		N (%)
Total	45 474 (100)		45 474 (100)
Total person-years from study start date to end of follow-up (1000s)	148.371	Alcohol status	
Age at first incident stroke (years)		Non-drinker	4914 (10.8)
40 to 59	7527 (16.6)	Current-drinker	29 044 (63.9)
60 to 74	14 578 (32.1)	Ex-drinker	6447 (14.2)
75 to 84	14 026 (30.8)	Missing	5069 (11.1)
Over 85	9343 (20.5)	BMI category WHO defined	
Sex		Underweight	1235 (2.7)
Male	23 196 (51)	Normal Weight	13 782 (30.3)
Female	22 278 (49.0)	Overweight	15 146 (33.3)
Ethnicity		Obese	10 318 (22.7)
Black	543 (1.2)	Missing	4993 (11.0)
Mixed/Other	520 (1.1)	Current smoker	
South Asian	885 (1.9)	No	35 461 (78.0)
White	43 526 (95.7)	Yes	8781 (19.3)
Dementia diagnosed in study period, excluding first 3 months		Missing	1232 (2.7)
No	40 850 (89.8)	Myocardial Infarction prior to stroke	
Yes	4624 (10.2)	No	41 887 (92.1)
Index of Multiple Deprivation (1 is least deprived)		Yes	3587 (7.9)
1	7273 (16.0)	Atrial fibrillation prior to stroke	
2	8407 (18.5)	No	39 449 (86.8)
3	9776 (21.5)	Yes	6025 (13.2)
4	9125 (20.1)	Uncontrolled diabetes prior to stroke	
5	10 893 (24.0)	No	42 857 (94.2)
Statin prescription 2 years prior to stroke		Yes	2617 (5.8)
No	29 599 (65.1)	Consultations per year in 3 years prior to stroke	
Yes	15 875 (34.9)	<10 per year	25 399 (55.9)
Uncontrolled HT (Sys≥140 and Dias≥90 mm Hg)—2 years prior to stroke		10–20 per year	12 962 (28.5)
No	40 710 (89.5)	20–30 per year	4496 (9.9)
Yes	4764 (10.5)	30–50 per year	2220 (4.9)
Immunosuppressive prescription 2 years prior to stroke		>50 per year	397 (0.9)
No	40 375 (88.8)	Patient has HES linkage	
Yes	5099 (11.2)	No	7089 (15.6)
Antiplatelets within 90 days of stroke onset		Yes	38 385 (84.4)
No	18 308 (40.3)		
Yes	27 166 (59.7)		

BMI, body mass index.

At incident stroke those from BAME groups were generally younger, had lower proportion of alcohol consumers, lower proportion with atrial fibrillation, and fewer consultations per year. They were also more deprived, had more prescription of statins, higher proportion overweight or obese, and higher proportion with uncontrolled diabetes (online supplementary E-table 1).

Overall, 4624 (10.2%) of these developed poststroke dementia- 1179 (25.5%) between 3 months and 1 year poststroke (early dementia), 2625 (56.8%) between 1 year to 5 years poststroke (late dementia), while 820 (17.7%) were diagnosed with dementia more than 5 years after stroke. Dementia diagnosis was associated with having an incident stroke at the age of 85 or more, those not prescribed statins, those who did not have uncontrolled hypertension, or uncontrolled diabetes, those who did not receive antiplatelets, the underweight, those not smoking currently, and those who had atrial-fibrillation in the univariate analysis (online supplementary E-table 2).

The overall crude rate of dementia was 31.4 per 1000 person-years for White, 19.0 per 1000 person-years for South Asian, 24.1 per 1000 person-years for Black, and 24.1 for Mixed/Other ethnicities.

### Ethnicity and poststroke dementia

Compared with those of White ethnicity the crude incidence (using age as an underlying timescale) of poststroke dementia diagnosis in the study period was 23% higher in South Asians (HR 1.23, 95% CI 0.96 to 1.56), 63% higher in those of Black ethnicity (HR 1.63, 95% CI 1.24 to 2.13), and the same in Mixed/Other ethnicities (HR 1.01, 95% CI 0.74 to 1.38). There was very strong evidence of an association between poststroke dementia and ethnicity with a p-value of 0.004 (table 2).

**Table 2:** Association between ethnicity and poststroke dementia results from Cox regression analyses, (n=45 474)

Ethnicity	Crude HR*	95% CI		Partially adjusted HR†	95% CI		Full model (without Uncontrolled HT and Diabetes)‡	95% CI	
		Lower	Upper		Lower	Upper		Lower	Upper
White	1			1			1		
South Asian	1.23	0.96	1.56	1.22	0.96	1.56	1.16	0.89	1.50
Black	1.63	1.24	2.13	1.60	1.22	2.09	1.42	1.05	1.93
Mixed/Other	1.01	0.74	1.38	1.01	0.74	1.38	0.97	0.68	1.38
P value§	0.004			0.006			0.130		

*Adjusting for age by using age as underlying timescale.

†Adjusting for age, sex, IMD.

‡Adjusting for above; prescriptions: statins, immunosuppressives, antiplatelets; alcohol, smoking; history before stroke: MI, atrial fibrillations, consultations per year.

§Obtained using likelihood ratio test.

IMD, Index of Multiple Deprivation.

After adjusting for age, sex, and IMD, the incidence of poststroke dementia diagnosis was 60% higher in the Black ethnic group (HR 1.60, 95% CI 1.22 to 2.09), while the HRs were 1.22 and 1.01 for South Asian and Mixed/Other ethnic groups, respectively. There was very strong evidence of an overall association with a p-value of 0.006 (table 2).

In the fully adjusted model the incidence of poststroke dementia diagnosis was 42% higher in the Black ethnic group (HR 1.42, 95% CI 1.05 to 1.93), while the HRs were 1.16 and 0.97 for South Asian and Mixed/Other ethnic groups, respectively. There was very weak evidence of an overall association between ethnicity and poststroke dementia (p=0.130) (see also supplementary E-tables 3-5).

**Table 3:** Association between Ethnicity and Poststroke Dementia results from Cox regression analyses using full model, by early, late, and very late dementia (n=45 474)

Ethnicity	Early dementia*	95%		Late dementia*	95%		Very late dementia*	95%	
		Lower	Upper		Lower	Upper		Lower	Upper
White	1			1			1		
South Asian	1.27	0.79	2.05	1.17	0.83	1.65	0.93	0.46	1.90
Black	1.33	0.71	2.49	1.62	1.10	2.37	0.93	0.38	2.25
Mixed/Other	0.88	0.42	1.86	0.88	0.54	1.44	1.33	0.66	2.67
P value†	0.634			0.103			0.886		

*Adjusting for age, sex, IMD; prescriptions: statins, immunosuppressives, antiplatelets; alcohol, smoking; history before stroke: MI, atrial fibrillations, consultations per year.

†Obtained using likelihood ratio test.

IMD, Index of Multiple Deprivation.

**Table 4:** Association between ethnicity and poststroke dementia results from Cox regression analyses, for HES linked data (n=38 385)

Ethnicity	Crude HR*	95% CI		Partially adjusted HR†	95% CI		Full model (without Uncontrolled HT and Diabetes)‡	95% CI	
		Lower	Upper		Lower	Upper		Lower	Upper
White	1			1			1		
South Asian	1.34	1.06	1.71	1.34	1.05	1.71	1.30	1.00	1.69
Black	1.67	1.27	2.21	1.64	1.24	2.16	1.46	1.07	2.01
Mixed/Other	1.11	0.81	1.53	1.11	0.81	1.53	1.12	0.78	1.62
P value*§	<0.001			0.001			0.037		

*Adjusting for age by using age as underlying timescale.

†Adjusting for age, sex, IMD.

‡Adjusting for above; prescriptions: statins, immunosuppressives, antiplatelets; alcohol, smoking; history before stroke: MI, atrial fibrillations, consultations per year.

§Obtained using likelihood ratio test.

IMD, Index of Multiple Deprivation.

Late dementia (one to 5 years after stroke) showed similar association with ethnicity overall in the fully adjusted model while there was no evidence of an association between poststroke dementia and early or very late dementia (table 3). Adjusting for potential mediators, uncontrolled hypertension and diabetes, gave results similar to the analysis excluding these (online supplementary files-E-table 6).

### Sensitivity analysis

Table 4 shows results after limiting the analysis to only patients with linked HES data.

After adjusting for age, sex, and IMD the incidence of poststroke dementia diagnosis was 34% higher in South Asians (HR 1.34, 95% CI 1.05 to 1.71) and 64% higher in the Black ethnic group (HR 1.64, 95% CI 1.24 to 2.16), while the HR was 1.11 for the Mixed/Other ethnic group. There was strong evidence of an overall association with a p-value of 0.001.

In the fully adjusted model the incidence of poststroke dementia diagnosis was 46% higher in the Black ethnic group (HR 1.46, 95% CI 1.07 to 2.01). There was some weak evidence an increased risk in South Asians (HR 1.30, 95% CI 1.00 to 1.69), while the HR was 1.12 for the Mixed/Other ethnic group. There was evidence of an overall association with a p-value of 0.037 (see also supplementary E-tables 8-12).

### Ethnic differences in premature exit from the study

We found no association between ethnicity and being excluded from the study in the first 3 months after stroke due to death (p-value 0.309), being diagnosed with dementia (p-value 0.430), all reasons excluding death and dementia diagnosis (p-value 0.567), or all reasons combined (p-value 0.577) (online supplementary material-E-table 7).

## Discussion

This UK population based cohort study found that stroke survivors of the Black ethnic group had a higher incidence of diagnosed dementia poststroke compared with the reference White ethnic group, and the confidence intervals did not include the null value. There was no evidence of an association with any other ethnic group, however the study was limited by small numbers. In those with linked HES data, from English practices, there was strong evidence of an overall association and those of Black ethnic group having a higher incidence of dementia diagnosis. There was also some evidence of an association with South Asians having a higher incidence of diagnosed dementia. Understanding the relationship between ethnicity and poststroke dementia could help in targeting healthcare interventions to those at most risk. These results are consistent with our hypothesis of higher incidence of diagnosed dementia in BAME groups.

Other studies looking at incidence of dementia diagnosis in the general population in the UK^10^ and the US^15^ by ethnicity presented similar findings for the Black ethnic group, while presenting lower incidence rates for South Asians compared with the White ethnic group. While we could not find published studies on poststroke dementia in the UK, studies from the US indicate higher cognitive decline in the Black ethnic group compared with the White ethnic group^16^ which is consistent with our findings, but higher incidence of stroke-related dementia diagnosis in South Asians compared with non-South Asians.^17^

Our data did not suggest any ethnic differences in those who were excluded from the study due to death, dementia diagnosis, or otherwise in the first 3 months after stroke (after adjustment for multiple sociodemographic and clinical confounders). It therefore seems unlikely that this exclusion would have affected our results.

While the Black ethnic group had higher uncontrolled hypertension and diabetes (online supplementary E-table 1) compared with the White ethnic group in the univariable comparison, when added to the full model these did not explain the association. There could be some misclassification in measuring these risk factors before stroke due to lower consultations per year for the Black ethnic group (online supplementary E-table 1). There are likely to be other mechanisms affecting this association, such as years of education, for which information is not collected in these data. This association could also be due to increased patient consultations poststroke leading to diagnosis of dementia in people which might have otherwise been missed. However, it is also possible that there was under-reporting of dementia symptoms^10 18^ in people from BAME communities due to stigma or cultural reasons. This could also be due to delay in accessing healthcare services by members of BAME communities unless they felt it was severe,^19^ or clinicians not recognising symptoms of dementia in BAME groups due to language barriers, or due to more family and community support which lead to delay in diagnosis of dementia.^20^ Hence, actual rates might be higher for those belonging to Black or South Asian ethnic groups than our findings. Clinicians should be aware of ethnic differences in dementia diagnosis in people with history of stroke, and poststroke period as risk period for dementia.

Strengths of our study include using CPRD, which is a large dataset of electronic health records from a real world population. Considered generalisable to the UK, it covers about 8% of the population.^21^ We used linked HES data to increase capture of diagnosed dementia. In the absence of other large studies, these data could inform current healthcare practice and future research. There are, however, some limitations.

We only had ethnicity data available on 75% of the potential study population and were unable to use data for the 14 918 people missing ethnicity information. We thought these were likely to be from the White ethnic group and hence not recorded. However, these data could potentially change our results if these were from BAME ethnic groups. We did not perform multiple imputation as we did not think ethnicity was missing at random. Although the HRs for poststroke dementia were lower in Mixed/Other ethnic group, there were relatively small numbers in this group and confidence intervals crossed the null value. We were working with a specific sub-group of stroke survivors, which might affect generalisability of findings. Additionally ethnic differences in dementia risk after stroke might be affected by factors such as stroke survival and behaviour after stroke for example, returning to country of origin. Nevertheless, we explored patterns of premature exit from the study and found no differences by ethnicity.

## Conclusions

The higher incidence of diagnosed dementia in the Black ethnic group shown in our study is consistent with our hypothesis. As far as we know, this is the first study to investigate poststroke dementia by ethnic groups and demonstrates that the Black ethnic group has increased risk. Although this difference is not explained by uncontrolled hypertension and diabetes in our study, there could be other reasons for this association. Further research is needed to better understand this relationship, including assessing the association using finer ethnic groupings, and across different settings and populations. Improved understanding of mechanisms could potentially lead to better and targeted diagnosis and care delivery for people of minority ethnic groups with dementia.

**What is already known on this subject**

- The UK has over 1.2 million stroke survivors. Stroke is a major risk factor for dementia.
- Along with other risk factors such as hypertension and diabetes, stroke is more common among Black, Asian and other ethnic minorities (BAME). As the UK population ages the proportion of elderly people from BAME is set to increase.
- Poststroke dementia is under-researched and has not been studied in a UK setting.

**What this study adds**

- Compared with the White ethnic group, there was evidence that the risk of a dementia diagnosis poststroke was higher in the Black ethnic group. While there was no strong evidence of an association with any other ethnic group the study was limited by small numbers.
- Further understanding of the mechanisms of this relationship could help target interventions at communities most at risk of dementia poststroke.
