# Supplementary material for: Ethnicity and risk of diagnosed dementia after stroke: a cohort study using the Clinical Practice Research Datalink
Source: J Epidemiol Community Health. 2019 Nov 7;74(2):114–9. doi: 10.1136/jech-2019-212825 (PMC6993022; doi:10.1136/jech-2019-212825)
Supplement: Supplementary data [file jech-2019-212825supp001.pdf]

Codelist 1

medcode	readterm	readcode	marker
1350	senile/presenile dementia	e00..12	Incident
1916	senile dementia	e00..11	Incident
1917	alzheimer's disease	f110.00	Incident
2731	cerebral atrophy	f11z.11	Incident
2882	senile or presenile psychoses nos	e00z.00	Incident
3591	huntington's chorea	f134.00	Cause_identified
3802	unspecified encephalopathy	f283.00	No
3979	hypertensive encephalopathy	g672.00	No
4357	[x] senile dementia nos	eu02z14	Incident
4501	wernicke's encephalopathy	c251.11	No
4693	[x] unspecified dementia	eu02z00	Incident
5644	anoxic - ischaemic encephalopathy	f281.11	No
5651	cerebral degeneration nos	f11z.00	Incident
5931	h/o: dementia	1461	Prevalent
6578	[x]vascular dementia	eu01.00	Incident
6709	[x]epileptic psychosis nos	eu05y11	No
7323	uncomplicated senile dementia	e000.00	Incident
7389	[x]acute / subacute confusional state, nonalcoholic	eu04.12	No
7572	lewy body disease	f116.00	Cause_identified
7664	[x]dementia in alzheimer's disease	eu00.00	Incident
8195	[x]alzheimer's dementia unspec	eu00z11	Incident
8634	multi infarct dementia	e004.11	Incident
8766	[x]symptomatic psychosis nos	eu0z.12	No
8934	[x]subcortical vascular dementia	eu01200	Incident
9509	[x]dementia in parkinson's disease	eu02300	Cause_identified
9565	[x]arteriosclerotic dementia	eu01.11	Incident
11107	wernicke's encephalopathy	c253.00	No
11136	pick's disease	f111.00	Cause_identified
11175	[x]multi-infarct dementia	eu01100	Incident
11379	[x]senile dementia,alzheimer's type	eu00112	Incident
11936	[x]mild cognitive disorder	eu05700	No
12621	[x]dementia in other diseases classified elsewhere	eu02.00	Cause_identified
12710	dementia annual review	6ab..00	Prevalent
15165	presenile dementia	e001.00	Incident
15261	cerebral ataxia	f11y100	No
16797	alzheimer's disease with early onset	f110000	Incident
18386	senile dementia with paranoia	e002000	Incident
19393	[x]vascular dementia, unspecified	eu01z00	Incident
19477	arteriosclerotic dementia	e004.00	Incident
20582	[x]post-traumatic brain syndrome	eu06212	No
21887	senile dementia with depression	e002100	Incident
22411	encephalopathy - hepatic	j622.11	No
23710	[x]personality and behav disorder brain dis dam and dysfunct	eu06.00	No
24387	[x]acute / subacute infective psychosis	eu04.13	No
24579	[x]postconcussional syndrome	eu06200	No
24581	cerebral degeneration due to vitamin b12 deficiency	f11x600	Cause_identified
25066	[x]delirium, not induced by alcohol+other psychoactive subs	eu04.00	No
25386	dementia in conditions ec	e041.00	Cause_identified
25704	[x]presenile dementia,alzheimer's type	eu00011	Incident
26270	[x]lewy body dementia	eu02500	Cause_identified
26323	[x]alcoholic dementia nos	eu10711	Cause_identified
27342	alcoholic dementia nos	e012.11	Cause_identified
27677	presenile dementia with depression	e001300	Incident
27759	[x] senile dementia, depressed or paranoid type	eu02z16	Incident
27935	[x] senile psychosis nos	eu02z15	Incident
27969	[x]oth mental disorder brain damag/dysfunction/physical disr	eu05.00	No
28402	[x]dementia in pick's disease	eu02000	Cause_identified
29386	[x]dementia in alzheimer's disease, unspecified	eu00z00	Incident
29512	senile degeneration of brain	f112.00	Incident
30032	presenile dementia with paranoia	e001200	Incident
30706	[x]dementia in alzheimer's dis, atypical or mixed type	eu00200	Incident
31016	[x]mixed cortical and subcortical vascular dementia	eu01300	Incident

Codelist 1

medcode	readterm	readcode	marker
31524	other cerebral degeneration nos	f11yz00	Incident
31877	[x]schizophrenia-like psychosis in epilepsy	eu05212	No
32057	alzheimer's disease with late onset	f110100	Incident
33353	reye's syndrome	f11y000	Cause_identified
34944	[x] primary degenerative dementia nos	eu02z13	Incident
34976	other cerebral degeneration	f11y.00	Incident
36748	alcoholic encephalopathy	f11x011	No
37014	[x]dementia in huntington's disease	eu02200	Cause_identified
37015	senile dementia with delirium	e003.00	Incident
37946	chronic alcoholic brain syndrome	e012000	Cause_identified
38286	jakob-creutzfeldt disease	a411.00	Cause_identified
38438	presenile dementia nos	e001z00	Incident
38678	[x]dementia in alzheimer's disease with late onset	eu00100	Incident
39337	[x]acute / subacute brain syndrome	eu04.11	No
41089	senile dementia with depressive or paranoid features nos	e002z00	Incident
41185	[x]dementia in human immunodef virus [hiv] disease	eu02400	Cause_identified
41744	toxic encephalopathy	f29y300	No
41950	[x]oth sp mental disord brain damag/dysfunction/physcal disd	eu05y00	No
42279	arteriosclerotic dementia nos	e004z00	Incident
42602	uncomplicated presenile dementia	e001000	Incident
43089	uncomplicated arteriosclerotic dementia	e004000	Incident
43292	arteriosclerotic dementia with depression	e004300	Incident
43346	[x]primary degen dementia of alzheimer's type, senile onset	eu00113	Incident
44592	cerebral degeneration other disease nos	f11xz00	Cause_identified
44674	senile dementia with depressive or paranoid features	e002.00	Incident
45602	myoclonic encephalopathy	f132200	No
46157	influenza with encephalopathy	h27y000	No
46488	[x]vascular dementia of acute onset	eu01000	Incident
46762	[x]alzheimer's disease type 1	eu00111	Incident
47555	cerebral degeneration due to alcoholism	f11x000	Cause_identified
47619	[x] presenile psychosis nos	eu02z12	Incident
47658	cerebral degeneration due to myxoedema	f11x500	Cause_identified
47802	bilirubin encephalopathy	q437000	No
48462	[x]limbic epilepsy personality	eu06013	No
48501	[x] presenile dementia nos	eu02z11	Incident
48531	cerebral degeneration due to jakob - creutzfeldt disease	f11x700	Cause_identified
49263	[x]dementia in alzheimer's disease with early onset	eu00000	Incident
49513	presenile dementia with delirium	e001100	Incident
49541	progressive multifocal leukoencephalopathy	a413.11	Cause_identified
49674	dementia monitoring first letter	9ou1.00	Prevalent
50069	[x]unspec mental disorder brain damag/dysfunction/physcal dr	eu05z00	No
50084	[x]postencephalitic syndrome	eu06100	No
51494	presbyophrenic psychosis	e00y.11	Incident
51665	[x]frontal lobe syndrome	eu06012	No
52394	[x]other delirium	eu04y00	No
52673	progressive multifocal leucoencephalopathy	a413.00	Cause_identified
52872	[x]oth organ personality behav disorders brain dis dam dysf	eu06y00	No
53446	[x]delirium superimposed on dementia	eu04100	Incident
53924	[x]delirium, unspecified	eu04z00	No
54106	[x]dementia in creutzfeldt-jakob disease	eu02100	Cause_identified
54505	other alcoholic dementia	e012.00	Cause_identified
54744	cerebral degeneration due to cerebrovascular disease	f11x200	Incident
55023	dementia monitoring	66h..00	Prevalent
55222	language disorder of dementia	zs7c500	Incident
55313	[x]other vascular dementia	eu01y00	Incident
55467	arteriosclerotic dementia with paranoia	e004200	Incident
55838	[x]predominantly cortical dementia	eu01111	Incident
56912	arteriosclerotic dementia with delirium	e004100	Incident
57183	mitochond encephalopathy, lact acidosis & strokelike episode	c315100	No
57993	[x]korsakov's psychosis, nonalcoholic	eu03.11	Cause_identified
59122	[x]other alzheimer's disease	fyu3000	Incident
60059	[x]primary degen dementia, alzheimer's type, presenile onset	eu00012	Incident

Codelist 1

medcode	readterm	readcode	marker
61528	[x]alzheimer's disease type 2	eu00013	Incident
62132	drug-induced dementia	e02y100	Cause_identified
63902	[x]unspec organ personality behav disorder brain dam dysfunc	eu06z00	No
64267	[x]dementia in other specified diseases classif elsewhere	eu02y00	Cause_identified
64416	[x]postcontusional syndrome	eu06211	No
65235	dementia monitoring telephone invite	9ou5.00	Prevalent
68125	[x]delirium not superimposed on dementia, so described	eu04000	No
68194	binswanger's encephalopathy	f21y211	Incident
70709	prion protein markers for creutzfeldt-jakob disease	4l49.00	Cause_identified
70957	cerebral degeneration due to neoplastic disease	f11x400	Cause_identified
83576	dementia monitoring second letter	9ou2.00	Prevalent
85853	dementia monitoring administration	9ou..00	Prevalent
89036	dementia monitoring third letter	9ou3.00	Prevalent
89037	dementia monitoring verbal invite	9ou4.00	Prevalent
89580		f1110a	No
96549	kendrick battery for detection of dementia in the elderly	zrv9.00	No
96860	cerebral degeneration in parkinson's disease	f11x900	Cause_identified
97422	cerebral degeneration in other disease ec	f11x.00	Cause_identified
97528	activity management for myalgic encephalopathy	8q1..11	No
99684	cerebral degeneration due to multifocal leucoencephalopathy	f11x800	Cause_identified
102189	arizona battery for communication disorders of dementia	zr1t.00	No
104155	suspected dementia	1ja2.00	Incident
106311	dementia care plan	8cmz.00	Prevalent
106446	nhs hlth chck rais awareness abt dementia and memory clinics	67df.00	No
108228	dementia advance care plan agreed	8csa.00	Prevalent
108268	review of dementia advance care plan	8cmg200	Prevalent
108391	dementia advance care plan declined	8iae000	Prevalent
109047	antipsychotic drug therapy for dementia	8bpa.00	Prevalent
109288	sporadic creutzfeldt-jakob disease	a411000	Cause_identified
109708	dementia care plan reviewed	8cmz100	Prevalent
109731	dementia care plan agreed	8cmz000	Prevalent
109737	dementia medication review	8bm0200	Prevalent
109786	dementia care plan declined	8cmz200	Prevalent
109790	dementia advance care plan	8cme000	Prevalent
110075	dementia advance care plan review declined	8iae200	Prevalent
110119	dementia leaflet given	8cet.00	Prevalent
110123	dementia care plan review declined	8cmz300	Prevalent
110729	[x]cognitive communication disorder	eu05800	No
111031	[x]postleucotomy syndrome	eu06015	No

Codelist 2

ICD10 code	description	marker
F00	Dementia in Alzheimer disease	Incident
F00.0	Dementia in Alzheimer disease with early onset	Incident
F00.1	Dementia in Alzheimer disease with late onset	Incident
F00.2	Dementia in Alzheimer disease, atypical or mixed type	Incident
F00.9	Dementia in Alzheimer disease, unspecified	Incident
F01	Vascular dementia	Incident
F01.1	Multi-infarct dementia	Incident
F01.2	Subcortical vascular dementia	Incident
F01.3	Mixed cortical and subcortical vascular dementia	Incident
F01.8	Other vascular dementia	Incident
F01.9	Vascular dementia, unspecified	Incident
F02	Dementia in other diseases classified elsewhere	Cause_identified
F02.0	Dementia in Pick disease	Cause_identified
F02.1	Dementia in Creutzfeldt-Jakob disease	Cause_identified
F02.2	Dementia in Huntington disease	Cause_identified
F02.3	Dementia in Parkinson disease	Cause_identified
F02.4	Dementia in human immunodeficiency virus [HIV] disease	Cause_identified
F02.8	Dementia in other specified diseases classified elsewhere	Cause_identified
F03	Unspecified dementia	Incident
F05.1	Delirium superimposed on dementia	Incident
F10.7	Mental and behavioural disorders due to use of alcohol	Cause_identified
G30	Alzheimer disease	Incident
G30.0	Alzheimer disease with early onset	Incident
G30.1	Alzheimer disease with late onset	Incident
G30.8	Other Alzheimer disease	Incident
G30.9	Alzheimer disease, unspecified	Incident
G31.0	Circumscribed brain atrophy	Incident
G31.1	Senile degeneration of brain, not elsewhere classified	Incident
G31.8	Other specified degenerative diseases of nervous system	Cause_identified
F01.0	Vascular dementia of acute onset	Incident

Codelist 3

medcode	readcode	prev	inc	readterm
569	g64..12	0	1	Infarction - cerebral
1298	g66..11	0	1	CVA unspecified
1469	g66..00	0	1	Stroke and cerebrovascular accident unspecified
1786	g60..00	0	1	Subarachnoid haemorrhage
3149	g64z.00	0	1	Cerebral infarction NOS
3535	g61z.00	0	1	Intracerebral haemorrhage NOS
4152	g631.12	0	1	Thrombosis, carotid artery
4240	g631.00	0	1	Carotid artery occlusion
5051	g61..00	0	1	Intracerebral haemorrhage
5185	g64z111	0	0	Lateral medullary syndrome
5363	g64..11	0	1	CVA - cerebral artery occlusion
5602	g64z.12	0	1	Cerebellar infarction
6116	g66..13	0	1	CVA - Cerebrovascular accident unspecified
6155	g64..13	0	1	Stroke due to cerebral arterial occlusion
6253	g66..12	0	1	Stroke unspecified
6960	g61..11	0	1	CVA - cerebrovascular accid due to intracerebral haemorrhage
7780	g667.00	0	1	Left sided CVA
7912	g614.00	0	1	Pontine haemorrhage
8443	g663.00	0	1	Brain stem stroke syndrome
8837	g64..00	0	1	Cerebral arterial occlusion
9696	g604.00	0	1	Subarachnoid haemorrhage from posterior communicating artery
9985	g64z200	0	1	Left sided cerebral infarction
10504	g64z300	0	1	Right sided cerebral infarction
12833	g668.00	0	1	Right sided CVA
13564	g613.00	0	1	Cerebellar haemorrhage
15019	g641.00	0	1	Cerebral embolism
15252	g64z.11	0	1	Brainstem infarction NOS
16517	g640.00	0	1	Cerebral thrombosis
17322	g664.00	0	1	Cerebellar stroke syndrome
17326	g60x.00	0	1	Subarachnoid haemorrh from intracranial artery, unspecif
18604	g61..12	0	1	Stroke due to intracerebral haemorrhage
18689	g660.00	0	1	Middle cerebral artery syndrome
19201	g61x100	0	1	Right sided intracerebral haemorrhage, unspecified
19260	g662.00	0	1	Posterior cerebral artery syndrome
19280	g661.00	0	1	Anterior cerebral artery syndrome
19412	g602.00	0	1	Subarachnoid haemorrhage from middle cerebral artery
20284	g62z.00	0	1	Intracranial haemorrhage NOS
23580	g60z.00	0	1	Subarachnoid haemorrhage NOS
23671	g63y000	0	1	Cerebral infarct due to thrombosis of precerebral arteries
24446	g63y100	0	1	Cerebral infarction due to embolism of precerebral arteries
25615	g64z000	0	1	Brainstem infarction
26424	g64z400	0	1	Infarction of basal ganglia
27975	g641000	0	1	Cerebral infarction due to embolism of cerebral arteries
28314	g61x000	0	1	Left sided intracerebral haemorrhage, unspecified
28807	s62..12	0	1	Subarachnoid haemorrhage following injury
29939	g600.00	0	1	Ruptured berry aneurysm
30045	g616.00	0	1	External capsule haemorrhage
30202	g617.00	0	1	Intracerebral haemorrhage, intraventricular
31060	g61x.00	0	1	Intracerebral haemorrhage in hemisphere, unspecified
31595	g610.00	0	1	Cortical haemorrhage
31805	g62..00	0	1	Other and unspecified intracranial haemorrhage

Codelist 3

medcode	readcode	prev	inc	readterm
32447	g630.00	0	1	Basilar artery occlusion
33499	g665.00	0	1	Pure motor lacunar syndrome
33543	g6x..00	0	1	Cerebrl infarctn due/unspcf occlusn or sten/cerebrl artrs
34758	g641.11	0	1	Cerebral embolus
36717	g640000	0	1	Cerebral infarction due to thrombosis of cerebral arteries
38304	s620.00	0	1	Closed traumatic subarachnoid haemorrhage
39344	g676000	0	1	Cereb infarct due cerebral venous thrombosis, nonpyogenic
40338	g611.00	0	1	Internal capsule haemorrhage
40758	g6w..00	0	1	Cereb infarct due unsp occlus/stenos precerebr arteries
40847	g632.00	0	1	Vertebral artery occlusion
41910	g605.00	0	1	Subarachnoid haemorrhage from basilar artery
42331	g603.00	0	1	Subarachnoid haemorrhage from anterior communicating artery
45781	g63..00	0	1	Precerebral arterial occlusion
46316	g612.00	0	1	Basal nucleus haemorrhage
47607	l440.11	0	1	CVA - cerebrovascular accident in the puerperium
47642	g64z100	0	0	Wallenberg syndrome
51326	g63y.00	0	1	Other precerebral artery occlusion
51759	g677000	0	1	Occlusion and stenosis of middle cerebral artery
51767	g666.00	0	1	Pure sensory lacunar syndrome
53745		0	1	[X]Other cerebral infarction
53810	gyu6200	0	1	[X]Other intracerebral haemorrhage
55602	g677300	0	1	Occlusion and stenosis of cerebellar arteries
56007	g601.00	0	1	Subarachnoid haemorrhage from carotid siphon and bifurcation
56279	l440.12	0	1	Stroke in the puerperium
57315	g618.00	0	1	Intracerebral haemorrhage, multiple localized
57495	g63..11	0	1	Infarction - precerebral
57527	g677100	0	1	Occlusion and stenosis of anterior cerebral artery
58545	s627.00	0	1	Traumatic subarachnoid haemorrhage
60692	g606.00	0	1	Subarachnoid haemorrhage from vertebral artery
62342	g615.00	0	1	Bulbar haemorrhage
65745	gyu6100	0	1	[X]Other subarachnoid haemorrhage
65770	g677200	0	1	Occlusion and stenosis of posterior cerebral artery
70536	g671000	0	1	Acute cerebrovascular insufficiency NOS
71274	g677400	0	1	Occlusion+stenosis of multiple and bilat cerebral arteries
71585	g63z.00	0	1	Precerebral artery occlusion NOS
90572		0	1	[X]Occlusion and stenosis of other precerebral arteries
91627		0	1	[X]Cerebrl infarctn due/unspcf occlusn or sten/cerebrl artrs
92036		0	1	[X]Occlusion and stenosis of other cerebral arteries
93459		0	1	[X]Other lacunar syndromes
94482		0	1	[X]Cereb infarct due unsp occlus/stenos precerebr arteries
96630	gyu6f00	0	1	[X]Intracerebral haemorrhage in hemisphere, unspecified
96717	s621.00	0	1	Open traumatic subarachnoid haemorrhage
98642	g633.00	0	1	Multiple and bilateral precerebral arterial occlusion
107440	g619.00	0	1	Lobar cerebral haemorrhage
108630		0	1	[X]Subarachnoid haemorrh from intracranial artery, unspecif
108668	gyu6000	0	1	[X]Subarachnoid haemorrhage from other intracranial arteries

Codelist 4

ICD10 code	description
I60	Subarachnoid haemorrhage
I60.0	Subarachnoid haemorrhage from carotid siphon and bifurcation
I60.1	Subarachnoid haemorrhage from middle cerebral artery
I60.2	Subarachnoid haemorrhage from anterior communicating artery
I60.3	Subarachnoid haemorrhage from posterior communicating artery
I60.4	Subarachnoid haemorrhage from basilar artery
I60.5	Subarachnoid haemorrhage from vertebral artery
I60.6	Subarachnoid haemorrhage from other intracranial arteries
I60.7	Subarachnoid haemorrhage from intracranial artery, unspecified
I60.8	Other subarachnoid haemorrhage
I60.9	Subarachnoid haemorrhage, unspecified
I61	Intracerebral haemorrhage
I61.0	Intracerebral haemorrhage in hemisphere, subcortical
I61.1	Intracerebral haemorrhage in hemisphere, cortical
I61.2	Intracerebral haemorrhage in hemisphere, unspecified
I61.3	Intracerebral haemorrhage in brain stem
I61.4	Intracerebral haemorrhage in cerebellum
I61.5	Intracerebral haemorrhage, intraventricular
I61.6	Intracerebral haemorrhage, multiple localized
I61.8	Other intracerebral haemorrhage
I61.9	Intracerebral haemorrhage, unspecified
I63	Cerebral infarction
I63.0	Cerebral infarction due to thrombosis of precerebral arteries
I63.1	Cerebral infarction due to embolism of precerebral arteries
I63.2	Cerebral infarction due to unspecified occlusion or stenosis of precerebral arteries
I63.3	Cerebral infarction due to thrombosis of cerebral arteries
I63.4	Cerebral infarction due to embolism of cerebral arteries
I63.5	Cerebral infarction due to unspecified occlusion or stenosis of cerebral arteries
I63.6	Cerebral infarction due to cerebral venous thrombosis, nonpyogenic
I63.8	Other cerebral infarction
I63.9	Cerebral infarction, unspecified
I64	Stroke, not specified as haemorrhage or infarction

E-Table 1

E-Table 1: Ethnicity and Post-stroke Dementia- univariate proportions by ethnicity

	Mixed/Other	Black	South Asian	White	Total	p-value
**N (%)**	520 (100)	543 (100)	885 (100)	43526 (100)	45474 (100)	
**Total person-years from study start date to end of follow-up (thousands)**	1.660	1.718	2.820	142.173	148.371	
**Age at first incident stroke**						
40 to 59 years	151 (29)	198 (36.5)	247 (27.9)	6931 (15.9)	7527 (16.6)	<0.001
60 to 74 years	171 (32.9)	190 (35)	346 (39.1)	13871 (31.9)	14578 (32.1)	
75 to 84 years	123 (23.7)	124 (22.8)	234 (26.4)	13545 (31.1)	14026 (30.8)	
Over 85 years	75 (14.4)	31 (5.7)	58 (6.6)	9179 (21.1)	9343 (20.5)	
**Sex**						
Male	272 (52.3)	271 (49.9)	485 (54.8)	22168 (50.9)	23196 (51)	0.121
Female	248 (47.7)	272 (50.1)	400 (45.2)	21358 (49.1)	22278 (49)	
**Early Dementia**						
No	512 (98.5)	528 (97.2)	866 (97.9)	42389 (97.4)	44295 (97.4)	0.375
Yes	8 (1.5)	15 (2.8)	19 (2.1)	1137 (2.6)	1179 (2.6)	
**Late Dementia**						
No	500 (96.2)	509 (93.7)	846 (95.6)	40994 (94.2)	42849 (94.2)	0.073
Yes	20 (3.8)	34 (6.3)	39 (4.4)	2532 (5.8)	2625 (5.8)	
**Very Late Dementia**						
No	508 (97.7)	538 (99.1)	875 (98.9)	42733 (98.2)	44654 (98.2)	0.139
Yes	12 (2.3)	5 (0.9)	10 (1.1)	793 (1.8)	820 (1.8)	
**Dementia diagnosed in Study Period, excl. 1st 3 months**						
No	480 (92.3)	489 (90.1)	817 (92.3)	39064 (89.7)	40850 (89.8)	0.02
Yes	40 (7.7)	54 (9.9)	68 (7.7)	4462 (10.3)	4624 (10.2)	
**Index of Multiple Deprivation (1 is least deprived)**						
1	74 (14.2)	26 (4.8)	96 (10.8)	7077 (16.3)	7273 (16)	<0.001
2	103 (19.8)	47 (8.7)	133 (15)	8124 (18.7)	8407 (18.5)	
3	98 (18.8)	110 (20.3)	236 (26.7)	9332 (21.4)	9776 (21.5)	
4	134 (25.8)	170 (31.3)	203 (22.9)	8618 (19.8)	9125 (20.1)	
5	111 (21.3)	190 (35)	217 (24.5)	10375 (23.8)	10893 (24)	
**Statin prescription 2 yrs prior to stroke**						
No	359 (69)	364 (67)	462 (52.2)	28414 (65.3)	29599 (65.1)	<0.001
Yes	161 (31)	179 (33)	423 (47.8)	15112 (34.7)	15875 (34.9)	
**Uncontrolled HT (Sys=140 & Dias>=90 mmHg)- 2yrs prior to stroke**						
No	473 (91)	456 (84)	808 (91.3)	38973 (89.5)	40710 (89.5)	<0.001
Yes	47 (9)	87 (16)	77 (8.7)	4553 (10.5)	4764 (10.5)	
**Immunosuppressive prescription 2yrs prior to stroke**						
No	485 (93.3)	492 (90.6)	781 (88.2)	38617 (88.7)	40375 (88.8)	0.005
Yes	35 (6.7)	51 (9.4)	104 (11.8)	4909 (11.3)	5099 (11.2)	
**Antiplatelets within 90 days of stroke onset**						
No	245 (47.1)	256 (47.1)	341 (38.5)	17466 (40.1)	18308 (40.3)	<0.001
Yes	275 (52.9)	287 (52.9)	544 (61.5)	26060 (59.9)	27166 (59.7)	

Shiekh SI, *et al. J Epidemiol Community Health* 2019; 74:114–119. doi: 10.1136/jech-2019-212825

E-Table 1

	Mixed/Other	Black	South Asian	White	Total	p-value
**N (%)**	520 (100)	543 (100)	885 (100)	43526 (100)	45474 (100)	
**Alcohol status**						
Non-drinker	124 (23.8)	137 (25.2)	424 (47.9)	4229 (9.7)	4914 (10.8)	<0.001
Current-drinker	242 (46.5)	240 (44.2)	243 (27.5)	28319 (65.1)	29044 (63.9)	
Ex-drinker	78 (15)	102 (18.8)	122 (13.8)	6145 (14.1)	6447 (14.2)	
missing	76 (14.6)	64 (11.8)	96 (10.8)	4833 (11.1)	5069 (11.1)	
**BMI category WHO defined**						
Underweight	19 (3.7)	9 (1.7)	23 (2.6)	1184 (2.7)	1235 (2.7)	<0.001
Normal Weight	141 (27.1)	131 (24.1)	281 (31.8)	13229 (30.4)	13782 (30.3)	
Overweight	174 (33.5)	170 (31.3)	343 (38.8)	14459 (33.2)	15146 (33.3)	
Obese	107 (20.6)	168 (30.9)	157 (17.7)	9886 (22.7)	10318 (22.7)	
missing	79 (15.2)	65 (12)	81 (9.2)	4768 (11)	4993 (11)	
**Current smoker**						
No	402 (77.3)	430 (79.2)	745 (84.2)	33,884 (77.8)	35461 (78.0)	<0.001
Yes	99 (19)	91 (16.8)	113 (12.8)	8478 (19.5)	8781 (19.3)	
missing	19 (3.7)	22 (4.1)	27 (3.1)	1164 (2.7)	1232 (2.7)	
**MI prior to stroke**						
No	485 (93.3)	529 (97.4)	791 (89.4)	40082 (92.1)	41887 (92.1)	<0.001
Yes	35 (6.7)	14 (2.6)	94 (10.6)	3444 (7.9)	3587 (7.9)	
**Afib prior to stroke**						
No	491 (94.4)	520 (95.8)	825 (93.2)	37613 (86.4)	39449 (86.8)	<0.001
Yes	29 (5.6)	23 (4.2)	60 (6.8)	5913 (13.6)	6025 (13.2)	
**Uncontrolled diabetes prior to stroke**						
No	471 (90.6)	479 (88.2)	732 (82.7)	41,175 (94.6)	42857 (94.2)	<0.001
Yes	49 (9.4)	64 (11.8)	153 (17.3)	2,351 (5.4)	2617 (5.8)	
**Consultations per year in 3yrs prior to stroke**						
< 10 per year	344 (66.2)	348 (64.1)	466 (52.7)	24241 (55.7)	25399 (55.9)	<0.001
10 - 20 per year	131 (25.2)	142 (26.2)	249 (28.1)	12440 (28.6)	12962 (28.5)	
20 - 30 per year	28 (5.4)	32 (5.9)	100 (11.3)	4336 (10)	4496 (9.9)	
30 - 50 per year	15 (2.9)	18 (3.3)	59 (6.7)	2128 (4.9)	2220 (4.9)	
> 50 per year	2 (0.4)	3 (0.6)	11 (1.2)	381 (0.9)	397 (0.9)	
**Consultations per year in follow-up after stroke**						
< 10 per year	148 (28.5)	155 (28.5)	196 (22.1)	9745 (22.4)	10244 (22.5)	<0.001
10 - 20 per year	173 (33.3)	179 (33)	269 (30.4)	14370 (33)	14991 (33)	
20 - 30 per year	97 (18.7)	120 (22.1)	185 (20.9)	9289 (21.3)	9691 (21.3)	
30 - 50 per year	76 (14.6)	70 (12.9)	170 (19.2)	7533 (17.3)	7849 (17.3)	
> 50 per year	26 (5)	19 (3.5)	65 (7.3)	2589 (5.9)	2699 (5.9)	
**Patient has HES linkage**						
No	90 (17.3)	63 (11.6)	130 (14.7)	6806 (15.6)	7089 (15.6)	0.039
Yes	430 (82.7)	480 (88.4)	755 (85.3)	36720 (84.4)	38385 (84.4)	

Shiekh SI, *et al. J Epidemiol Community Health* 2019; 74:114–119. doi: 10.1136/jech-2019-212825

E-Table 2

E-Table 2: Ethnicity and Post-stroke Dementia- % diagnosed with dementia during study period			
	**Yes (%)**	**Total**	**p-value**
**N**	4624 (10.2)	45,474	
**Total person-years from study start date to end of follow-u**	11.996 (8.1)	148.371	
**Age at first incident stroke**			<0.001
40 to 59 years	83 (1.1)	7527	
60 to 74 years	859 (5.9)	14578	
75 to 84 years	2024 (14.4)	14,026	
Over 85 years	1658 (17.7)	9,343	
**Sex**			<0.001
Male	2017 (8.7)	23,196	
Female	2607 (11.7)	22,278	
**Ethnicity**			0.020
White	4462 (10.3)	43,526	
South Asian	68 (7.7)	885	
Black	54 (9.9)	543	
Mixed/Other	40 (7.7)	520	
**Index of Multiple Deprivation (1 is least deprived)**			0.236
1	763 (10.5)	7273	
2	882 (10.5)	8407	
3	998 (10.2)	9776	
4	934 (10.2)	9125	
5	1047 (9.6)	10,893	
**Statin prescription 2 yrs prior to stroke**			<0.001
No	3161 (10.7)	29,599	
Yes	1463 (9.2)	15,875	
**Uncontrolled HT (Sys=140 & Dias>=90 mmHg)- 2yrs prior to stroke**			<0.001
No	4243 (10.4)	40,717	
Yes	381 (8)	4757	
**Immunosuppressive prescription 2yrs prior to stroke**			<0.001
No	4193 (10.4)	40,375	
Yes	431 (8.5)	5099	
**Antiplatelets within 90 days of stroke onset**			<0.001
No	2127 (11.6)	18308	
Yes	2497 (9.2)	27166	
**Alcohol status**			<0.001
Non-drinker	597 (12.1)	4914	
Current-drinker	2499 (8.6)	29,044	
Ex-drinker	676 (10.5)	6447	
missing	852 (16.8)	5069	
**BMI category WHO defined**			<0.001
Underweight	199 (16.1)	1235	
Normal Weight	1532 (11.1)	13,782	
Overweight	1397 (9.2)	15,146	
Obese	622 (6)	10318	
missing	874 (17.5)	4993	

E-Table 2

	Yes (%)	Total	p-value
**Current smoker**			
No	3806 (10.7)	35,461	<0.001
Yes	552 (6.3)	8781	
missing	266 (21.6)	1232	
**MI prior to stroke**			
No	4232 (10.1)	41,887	0.117
Yes	392 (10.9)	3587	
**Afib prior to stroke**			
No	3859 (9.8)	39,449	<0.001
Yes	765 (12.7)	6025	
**Uncontrolled diabetes prior to stroke**			
No	4424 (10.3)	42,857	0.126
Yes	200 (7.6)	2617	
**Consultations per year in 3yrs prior to stroke**			
< 10 per year	2105 (10.9)	19,315	<0.001
10 - 20 per year	1243 (9.1)	13,704	
20 - 30 per year	714 (10.2)	7011	
30 - 50 per year	454 (10.2)	4439	
> 50 per year	108 (10.7)	1005	
**Consultations per year in follow-up after stroke**			
< 10 per year	992 (17.4)	5709	<0.001
10 - 20 per year	858 (7.3)	11694	
20 - 30 per year	992 (9.2)	10780	
30 - 50 per year	1204 (10.4)	11,622	
> 50 per year	578 (10.2)	5669	
**Patient has HES linkage**			
No	420 (5.9)	7089	<0.001
Yes	4204 (11)	38,385	

Shiekh SI, *et al. J Epidemiol Community Health* 2019; 74:114–119. doi: 10.1136/jech-2019-212825

E-Table 3

E-Table 3: Ethnicity and Post-stroke Dementia Cox regression crude analysis (Adjusted for Age in stset origin)

	Crude Univariate HR	95% CI		lrtest p-value	N
		Lower	Upper		
**Ethnicity**				0.004	45474
White	1				
South Asian	1.226	0.965	1.560		
Black	1.629	1.245	2.132		
Mixed/Other	1.009	0.739	1.377		
**Sex**				0.203	45474
Male	1				
Female	1.039	0.979	1.103		
**Index of Multiple Deprivation (1 is least deprived)**				0.061	45474
1	1				
2	1.038	0.942	1.144		
3	1.041	0.948	1.144		
4	1.111	1.010	1.223		
5	1.126	1.026	1.237		
**Statin prescription 2 yrs prior to stroke**				<0.001	45474
No	1				
Yes	0.898	0.844	0.956		
**Uncontrolled HT (Sys=140 & Dias>=90 mmHg)- 2yrs prior to stroke**				0.002	45474
No	1				
Yes	0.852	0.767	0.946		
**Immunosuppressive prescription 2yrs prior to stroke**				0.131	45474
No	1				
Yes	0.927	0.839	1.024		
**Antiplatelets within 90 days of stroke onset**				<0.001	45474
No	1				
Yes	0.683	0.644	0.723		
**Alcohol status**				<0.001	40405
Non-drinker	1				
Current-drinker	0.772	0.706	0.844		
Ex-drinker	0.896	0.802	1.000		

Shiekh SI, *et al. J Epidemiol Community Health* 2019; 74:114–119. doi: 10.1136/jech-2019-212825

E-Table 3

	Crude Univariate HR	95% CI		lrtest p-value	N
		Lower	Upper		
**BMI category WHO defined**				<0.001	40481
Underweight	1.610	1.388	1.867		
Normal Weight	1				
Overweight	0.877	0.816	0.944		
Obese	0.746	0.679	0.820		
**Current smoker**				0.057	44242
No	1				
Yes	1.094	0.998	1.199		
**MI prior to stroke**				0.608	45474
No	1				
Yes	1.028	0.926	1.140		
**Afib prior to stroke**				0.127	45474
No	1				
Yes	1.063	0.983	1.150		
**Uncontrolled diabetes prior to stroke**				0.121	45474
No	1				
Yes	1.121	0.973	1.292		
**Consultations per year in 3yrs prior to stroke**				<0.001	45474
< 10 per year	1				
10 - 20 per year	0.841	0.786	0.900		
20 - 30 per year	0.941	0.850	1.041		
30 - 50 per year	1.137	0.991	1.304		
> 50 per year	1.545	1.135	2.104		

Shiekh SI, *et al. J Epidemiol Community Health* 2019; 74:114–119. doi: 10.1136/jech-2019-212825

E-Table 4

E-Table 4: Ethnicity and Post-stroke Dementia Cox regression analysis- partially adjusted

	Adjusted HR for age, sex, imd	95% CI			
		Lower	Upper	lrtest p-value	N
**Ethnicity***				0.006	45474
White	1				
South Asian	1.223	0.962	1.556		
Black	1.597	1.220	2.090		
Mixed/Other	1.008	0.738	1.376		
**Sex***				0.2259	45474
Male	1				
Female	1.037	0.978	1.100		
**Index of Multiple Deprivation (1 is least deprived)***				0.091	45474
1	1				
2	1.037	0.941	1.142		
3	1.037	0.943	1.139		
4	1.105	1.004	1.216		
5	1.118	1.018	1.228		
**Statin prescription 2 yrs prior to stroke**				<0.001	45474
No	1				
Yes	0.895	0.840	0.953		
**Uncontrolled HT (Sys=140 & Dias>=90 mmHg)- 2yrs prior to stroke**				0.002	45474
No	1				
Yes	0.852	0.767	0.946		
**Immunosuppressive prescription 2yrs prior to stroke**				0.109	45474
No	1				
Yes	0.923	0.836	1.019		
**Antiplatelets within 90 days of stroke onset**				<0.001	45474
No	1.000				
Yes	0.681	0.643	0.722		
**Alcohol status**				<0.001	40405
Non-drinker	1				
Current-drinker	0.785	0.715	0.862		
Ex-drinker	0.901	0.806	1.007		

Shiekh SI, *et al. J Epidemiol Community Health* 2019; 74:114–119. doi: 10.1136/jech-2019-212825

E-Table 4

	Adjusted HR for age, sex, imd	95% CI			
		Lower	Upper	lrtest p-value	N
**BMI category WHO defined**				<0.001	40481
Underweight	1.588	1.369	1.843		
Normal Weight	1				
Overweight	0.876	0.814	0.943		
Obese	0.740	0.673	0.813		
**Current smoker**				0.0787	44242
No	1				
Yes	1.087	0.991	1.192		
**MI prior to stroke**				0.5248	45474
No	1				
Yes	1.035	0.932	1.148		
**Afib prior to stroke**				0.0861	45474
No	1				
Yes	1.071	0.991	1.159		
**Uncontrolled diabetes prior to stroke**				0.199	45474
No	1				
Yes	1.100	0.953	1.268		
**Consultations per year in 3yrs prior to stroke**				<0.001	45474
< 10 per year	1				
10 - 20 per year	0.840	0.785	0.899		
20 - 30 per year	0.942	0.851	1.043		
30 - 50 per year	1.135	0.989	1.302		
> 50 per year	1.534	1.127	2.089		

* adjusted with each other

Shiekh SI, *et al. J Epidemiol Community Health* 2019; 74:114–119. doi: 10.1136/jech-2019-212825

E-Table 5

E-Table 5: Ethnicity and Post-stroke Dementia Cox multivariable regression analysis without potential mediators uncontrolled HT and diabetes (lrtest p-value 0.1296)

	Full model without Uncontrolled HT and Diabetes	95% CI	
		Lower	Upper
**Ethnicity**			
White	1		
South Asian	1.158	0.893	1.503
Black	1.422	1.046	1.931
Mixed/Other	0.965	0.677	1.376
**Sex**			
Male	1		
Female	0.998	0.933	1.068
**Index of Multiple Deprivation (1 is least deprived)**			
1	1		
2	0.971	0.871	1.084
3	1.002	0.901	1.114
4	1.103	0.991	1.227
5	1.108	0.998	1.229
**Statin prescription 2 yrs prior to stroke**			
No	1		
Yes	1.013	0.942	1.089
**Immunosuppressive prescription 2yrs prior to stroke**			
No	1		
Yes	0.955	0.858	1.062
**Antiplatelets within 90 days of stroke onset**			
No	1.000		
Yes	0.794	0.741	0.850
**Alcohol status**			
Non-drinker	1		
Current-drinker	0.803	0.731	0.883
Ex-drinker	0.917	0.819	1.026
**Current smoker**			
No	1		
Yes	1.161	1.054	1.280
**MI prior to stroke**			
No	1		
Yes	1.103	0.984	1.236
**Afib prior to stroke**			
No	1		
Yes	1.033	0.945	1.129

Shiekh SI, *et al. J Epidemiol Community Health* 2019; 74:114–119. doi: 10.1136/jech-2019-212825

E-Table 5

	Full model without Uncontrolled HT and Diabetes	95% CI	
		Lower	Upper
**Consultations per year in 3yrs prior to stroke**			
< 10 per year	1		
10 - 20 per year	1.010	0.935	1.090
20 - 30 per year	1.122	1.004	1.254
30 - 50 per year	1.361	1.173	1.579
> 50 per year	1.827	1.331	2.507

Shiekh SI, *et al. J Epidemiol Community Health* 2019; 74:114–119. doi: 10.1136/jech-2019-212825

E-Table 6

E-Table 6: Ethnicity and Post-stroke Dementia Cox multivariable regression analysis with potential mediators uncontrolled HT and diabetes (lrtest p-value 0.1602)

	Full model with Uncontrolled HT and Diabetes	95% CI	
		Lower	Upper
**Ethnicity**			
White	1		
South Asian	1.141	0.879	1.482
Black	1.405	1.034	1.909
Mixed/Other	0.962	0.675	1.371
**Sex**			
Male	1		
Female	1.000	0.935	1.070
**Index of Multiple Deprivation (1 is least deprived)**			
1	1		
2	0.971	0.870	1.083
3	1.001	0.900	1.112
4	1.100	0.989	1.224
5	1.106	0.997	1.226
**Statin prescription 2 yrs prior to stroke**			
No	1		
Yes	1.003	0.933	1.079
**Uncontrolled HT (Sys=140 & Dias>=90 mmHg)- 2yrs prior to stroke**			
No	1		
Yes	0.940	0.843	1.049
**Immunosuppressive prescription 2yrs prior to stroke**			
No	1		
Yes	0.957	0.860	1.065
**Antiplatelets within 90 days of stroke onset**			
No	1		
Yes	0.793	0.740	0.849
**Alcohol status**			
Non-drinker	1		
Current-drinker	0.807	0.735	0.887
Ex-drinker	0.917	0.819	1.026

Shiekh SI, *et al. J Epidemiol Community Health* 2019; 74:114–119. doi: 10.1136/jech-2019-212825

E-Table 6

	Full model with Uncontrolled HT and Diabetes	95% CI	
		Lower	Upper
**Smoking status**			
Non-smoker	1		
Current-smoker	1.163	1.056	1.282
Ex-smoker			
**MI prior to stroke**			
No	1		
Yes	1.101	0.982	1.234
**Afib prior to stroke**			
No	1		
Yes	1.035	0.947	1.131
**Diabetes prior to stroke**			
No	1		
Yes	1.170	1.009	1.357
**Consultations per year in follow-up after stroke**			
< 10 per year	1		
10 - 20 per year	1.005	0.931	1.085
20 - 30 per year	1.111	0.994	1.242
30 - 50 per year	1.343	1.157	1.559
> 50 per year	1.794	1.307	2.464

Shiekh SI, *et al. J Epidemiol Community Health* 2019; 74:114–119. doi: 10.1136/jech-2019-212825

E-Table 7

E-Table 7: Ethnicity and premature exit from study in the first three months after stroke due to different causes using full Cox regression model (N=57930)**

Ethnicity	Death in first three months (n=8085)	95% CI		Dementia diagnosed in first three months (n=1830)	95% CI		Censoring in first three months (excluding death/dementia) (n=2597)	95% CI		Censoring in first three months (all reasons) (n=10821)	95% CI	
		Lower	Upper		Lower	Upper		Lower	Upper		Lower	Upper
lrtest p-value	0.309			0.430			0.567			0.577		
White	1			1			1			1		
South Asian	0.895	0.706	1.135	1.224	0.682	2.197	0.940	0.675	1.310	0.908	0.749	1.102
Black	0.961	0.730	1.264	1.716	0.884	3.334	1.239	0.862	1.782	1.043	0.838	1.299
Mixed/Other	0.797	0.603	1.054	1.225	0.580	2.588	1.167	0.803	1.696	0.901	0.720	1.127

** adjusting for age, sex, imd; prescriptions: statins, immunosuppressives, antiplatelets; alcohol, smoking; history before stroke: MI, Atrial fibrillations, consultations per year

Shiekh SI, *et al. J Epidemiol Community Health* 2019; 74:114–119. doi: 10.1136/jech-2019-212825

E-Table 8

E-Table 8: Ethnicity and Post-stroke Dementia- univariate proportions by ethnicity, for hes linked data

	Mixed/Other	Black	South Asian	White	Total	p-value
**N**	430 ()	480 ()	755 ()	36720 ()	38385 (100)	
**Total person-years from**	1.350	1.519	2.365	116.588	121.822	
**Age at first incident stroke**						
40 to 59 years	130 (30.2)	185 (38.5)	208 (27.5)	5569 (15.2)	6092 (15.9)	<0.001
60 to 74 years	137 (31.9)	161 (33.5)	293 (38.8)	11348 (30.9)	11939 (31.1)	
75 to 84 years	105 (24.4)	106 (22.1)	201 (26.6)	11541 (31.4)	11953 (31.1)	
Over 85 years	58 (13.5)	28 (5.8)	53 (7)	8262 (22.5)	8401 (21.9)	
**Sex**						
Male	224 (52.1)	242 (50.4)	415 (55)	18550 (50.5)	19431 (50.6)	0.100
Female	206 (47.9)	238 (49.6)	340 (45)	18170 (49.5)	18954 (49.4)	
**Early Dementia**						
No	423 (98.4)	466 (97.1)	736 (97.5)	35684 (97.2)	37309 (97.2)	0.480
Yes	7 (1.6)	14 (2.9)	19 (2.5)	1036 (2.8)	1076 (2.8)	
**Late Dementia**						
No	411 (95.6)	448 (93.3)	717 (95)	34416 (93.7)	35992 (93.8)	0.208
Yes	19 (4.4)	32 (6.7)	38 (5)	2304 (6.3)	2393 (6.2)	
**Very Late Dementia**						
No	418 (97.2)	475 (99)	745 (98.7)	36012 (98.1)	37650 (98.1)	0.162
Yes	12 (2.8)	5 (1)	10 (1.3)	708 (1.9)	735 (1.9)	
**Dementia diagnosed in**						
No	392 (91.2)	429 (89.4)	688 (91.1)	32672 (89)	34181 (89)	0.135
Yes	38 (8.8)	51 (10.6)	67 (8.9)	4048 (11)	4204 (11)	
**Index of Multiple**						
1	48 (11.2)	26 (5.4)	84 (11.1)	5921 (16.1)	6079 (15.8)	<0.001
2	89 (20.7)	41 (8.5)	121 (16)	7220 (19.7)	7471 (19.5)	
3	90 (20.9)	84 (17.5)	218 (28.9)	7580 (20.6)	7972 (20.8)	
4	102 (23.7)	153 (31.9)	160 (21.2)	7442 (20.3)	7857 (20.5)	
5	101 (23.5)	176 (36.7)	172 (22.8)	8557 (23.3)	9006 (23.5)	
**Statin prescription 2 yrs**						
No	307 (71.4)	329 (68.5)	416 (55.1)	24657 (67.1)	25709 (67)	<0.001
Yes	123 (28.6)	151 (31.5)	339 (44.9)	12063 (32.9)	12676 (33)	
**Uncontrolled HT (Sys=140 &**						
No	391 (90.9)	399 (83.1)	691 (91.5)	32921 (89.7)	34402 (89.6)	<0.001
Yes	39 (9.1)	81 (16.9)	64 (8.5)	3799 (10.3)	3983 (10.4)	
**Immunosuppressive**						
No	404 (94)	437 (91)	662 (87.7)	32718 (89.1)	34221 (89.2)	0.003
Yes	26 (6)	43 (9)	93 (12.3)	4002 (10.9)	4164 (10.8)	

E-Table 8

	Mixed/Other	Black	South Asian	White	Total	p-value
**N**	430 ()	480 ()	755 ()	36720 ()	38385 (100)	
**Antiplatelets within 90 days**						<0.001
No	208 (48.4)	238 (49.6)	305 (40.4)	15427 (42)	16178 (42.1)	
Yes	222 (51.6)	242 (50.4)	450 (59.6)	21293 (58)	22207 (57.9)	
**Alcohol status**						<0.001
Non-drinker	104 (24.2)	122 (25.4)	340 (45)	3601 (9.8)	4167 (10.9)	
Current-drinker	193 (44.9)	212 (44.2)	219 (29)	23541 (64.1)	24165 (63)	
Ex-drinker	62 (14.4)	83 (17.3)	104 (13.8)	4987 (13.6)	5236 (13.6)	
missing	71 (16.5)	63 (13.1)	92 (12.2)	4591 (12.5)	4817 (12.5)	
**BMI category WHO defined**						<0.001
Underweight	16 (3.7)	8 (1.7)	23 (3)	1019 (2.8)	1066 (2.8)	
Normal Weight	125 (29.1)	108 (22.5)	240 (31.8)	11180 (30.4)	11653 (30.4)	
Overweight	136 (31.6)	148 (30.8)	288 (38.1)	11983 (32.6)	12555 (32.7)	
Obese	82 (19.1)	151 (31.5)	126 (16.7)	8000 (21.8)	8359 (21.8)	
missing	71 (16.5)	65 (13.5)	78 (10.3)	4538 (12.4)	4752 (12.4)	
**Smoking status**						<0.001
No	330 (76.7)	373 (77.7)	630 (83.4)	28,751 (78.3)	30084 (78.4)	
Yes	81 (18.8)	85 (17.7)	98 (13)	6809 (18.5)	7073 (18.4)	
missing	19 (4.4)	22 (4.6)	27 (3.6)	1160 (3.2)	1228 (3.2)	
**MI prior to stroke**						<0.001
No	401 (93.3)	467 (97.3)	675 (89.4)	33884 (92.3)	35427 (92.3)	
Yes	29 (6.7)	13 (2.7)	80 (10.6)	2836 (7.7)	2958 (7.7)	
**Afib prior to stroke**						<0.001
No	409 (95.1)	460 (95.8)	706 (93.5)	31686 (86.3)	33261 (86.7)	
Yes	21 (4.9)	20 (4.2)	49 (6.5)	5034 (13.7)	5124 (13.3)	
**Uncontrolled diabetes prior**						<0.001
No	393 (91.4)	423 (88.1)	640 (84.8)	34914 (95.1)	36370 (94.8)	
Yes	37 (8.6)	57 (11.9)	115 (15.2)	1806 (4.9)	2015 (5.2)	
**Consultations per year in**						<0.001
< 10 per year	299 (69.5)	315 (65.6)	414 (54.8)	21088 (57.4)	22116 (57.6)	
10 - 20 per year	99 (23)	119 (24.8)	201 (26.6)	10228 (27.9)	10647 (27.7)	
20 - 30 per year	21 (4.9)	28 (5.8)	81 (10.7)	3489 (9.5)	3619 (9.4)	
30 - 50 per year	9 (2.1)	15 (3.1)	50 (6.6)	1643 (4.5)	1717 (4.5)	
> 50 per year	2 (0.5)	3 (0.6)	9 (1.2)	272 (0.7)	286 (0.7)	
**Consultations per year in**						<0.001
< 10 per year	127 (29.5)	140 (29.2)	174 (23)	8595 (23.4)	9036 (23.5)	
10 - 20 per year	146 (34)	158 (32.9)	226 (29.9)	12015 (32.7)	12545 (32.7)	
20 - 30 per year	79 (18.4)	104 (21.7)	151 (20)	7721 (21)	8055 (21)	
30 - 50 per year	60 (14)	60 (12.5)	146 (19.3)	6222 (16.9)	6488 (16.9)	
> 50 per year	18 (4.2)	18 (3.8)	58 (7.7)	2167 (5.9)	2261 (5.9)	

Shiekh SI, *et al. J Epidemiol Community Health* 2019; 74:114–119. doi: 10.1136/jech-2019-212825

E-Table 9

E-Table 9: Ethnicity and Post-stroke Dementia- % diagnosed with dementia during study period, for hes linked data			
	**Yes (%)**	**Total**	**p-value**
**N**	4204 (11)	38,385	
**Total person-years from study start date to end of follow-up (thousands)**	10.842 (8.9)	122	
**Age at first incident stroke**			<0.001
40 to 59 years	70 (1.1)	6092	
60 to 74 years	745 (6.2)	11939	
75 to 84 years	1821 (15.2)	11,953	
Over 85 years	1568 (18.7)	8,401	
**Sex**			<0.001
Male	1829 (9.4)	19,431	
Female	2375 (12.5)	18,954	
**eth5**			0.135
White	4048 (11)	36,720	
South Asian	67 (8.9)	755	
Black	51 (10.6)	480	
Mixed/Other	38 (8.8)	430	
**Index of Multiple Deprivation (1 is least deprived)**			0.507
1	691 (11.4)	6079	
2	839 (11.2)	7471	
3	871 (10.9)	7972	
4	854 (10.9)	7857	
5	949 (10.5)	9,006	
**Statin prescription 2 yrs prior to stroke**			<0.001
No	2964 (11.5)	25,709	
Yes	1240 (9.8)	12,676	
**Uncontrolled HT (Sys=140 & Dias>=90 mmHg)- 2yrs prior to stroke**			<0.001
No	3862 (11.2)	34,402	
Yes	342 (8.6)	3983	
**Immunosuppressive prescription 2yrs prior to stroke**			<0.001
No	3829 (11.2)	34,221	
Yes	375 (9)	4164	
**Antiplatelets within 90 days of stroke onset**			<0.001
No	2011 (12.4)	16178	
Yes	2193 (9.9)	22207	
**Alcohol status**			<0.001
Non-drinker	541 (13)	4167	
Current-drinker	2226 (9.2)	24,165	
Ex-drinker	594 (11.3)	5236	
missing	843 (17.5)	4817	
**BMI category WHO defined**			<0.001
Underweight	186 (17.4)	1066	
Normal Weight	1387 (11.9)	11,653	
Overweight	1240 (9.9)	12,555	
Obese	529 (6.3)	8359	
missing	862 (18.1)	4752	

Shiekh SI, *et al. J Epidemiol Community Health* 2019; 74:114–119. doi: 10.1136/jech-2019-212825

E-Table 9

	Yes (%)	Total	p-value
**Smoking status**			
Non-smoker	3453 (11.5)	30,084	<0.001
Current-smoker	485 (6.9)	7073	
missing	266 (21.7)	1228	
**MI prior to stroke**			
No	3862 (10.9)	35,427	0.269
Yes	342 (11.6)	2958	
**Afib prior to stroke**			
No	3524 (10.6)	33,261	<0.001
Yes	680 (13.3)	5124	
**Uncontrolled diabetes prior to stroke**			
No	4036 (11.1)	36,370	0.250
Yes	168 (8.3)	2015	
**Consultations per year in 3yrs prior to stroke**			
< 10 per year	2557 (11.6)	22,116	<0.001
10 - 20 per year	1062 (10)	10,647	
20 - 30 per year	377 (10.4)	3619	
30 - 50 per year	177 (10.3)	1717	
> 50 per year	31 (10.8)	286	
**Consultations per year in follow-up after stroke**			
< 10 per year	1313 (14.5)	9036	<0.001
10 - 20 per year	1170 (9.3)	12545	
20 - 30 per year	786 (9.8)	8055	
30 - 50 per year	693 (10.7)	6,488	
> 50 per year	242 (10.7)	2261	

Shiekh SI, *et al. J Epidemiol Community Health* 2019; 74:114–119. doi: 10.1136/jech-2019-212825

E-Table 10

E-Table 10: Ethnicity and Post-stroke Dementia Cox regression crude analysis, for hes linked data (Adjusted for Age in stset origin)

	Crude Univariate HR	95% CI			
		Lower	Upper	lrtest p-value	N
**Ethnicity**				<0.001	38385
White	1				
South Asian	1.345	1.055	1.714		
Black	1.672	1.268	2.206		
Mixed/Other	1.115	0.810	1.535		
**Sex**				0.406	38385
Male	1				
Female	1.027	0.965	1.092		
**Index of Multiple Deprivation (1 is least deprived)**				0.087	38385
1	1				
2	1.018	0.920	1.126		
3	1.037	0.938	1.146		
4	1.077	0.974	1.190		
5	1.131	1.025	1.247		
**Statin prescription 2 yrs prior to stroke**				<0.001	38385
No	1				
Yes	0.888	0.830	0.949		
**Uncontrolled HT (Sys=140 & Dias>=90 mmHg)- 2yrs prior to stroke**				<0.001	38385
No	1				
Yes	0.816	0.731	0.912		
**Immunosuppressive prescription 2yrs prior to stroke**				0.087	38385
No	1				
Yes	0.913	0.821	1.015		
**Antiplatelets within 90 days of stroke onset**				<0.001	38385
No	1				
Yes	0.690	0.650	0.734		
**Alcohol status**				<0.001	33568
Non-drinker	1				
Current-drinker	0.772	0.703	0.849		
Ex-drinker	0.901	0.802	1.012		

Shiekh SI, *et al. J Epidemiol Community Health* 2019; 74:114–119. doi: 10.1136/jech-2019-212825

E-Table 10

	Crude Univariate HR	95% CI		lrtest p-value	N
		Lower	Upper		
**BMI category WHO defined**				<0.001	33633
Underweight	1.608	1.379	1.875		
Normal Weight	1				
Overweight	0.881	0.816	0.952		
Obese	0.738	0.666	0.817		
**Current smoker**				0.015	37157
No	1				
Yes	1.131	1.025	1.247		
**MI prior to stroke**				0.917	38385
No	1				
Yes	1.006	0.901	1.124		
**Afib prior to stroke**				0.480	38385
No	1				
Yes	1.030	0.949	1.119		
**Uncontrolled diabetes prior to stroke**				0.230	38385
No	1				
Yes	1.101	0.943	1.285		
**Consultations per year in 3yrs prior to stroke**				<0.001	38385
< 10 per year	1				
10 - 20 per year	0.834	0.777	0.896		
20 - 30 per year	0.946	0.849	1.054		
30 - 50 per year	1.127	0.968	1.313		
> 50 per year	1.535	1.077	2.188		

Shiekh SI, *et al. J Epidemiol Community Health* 2019; 74:114–119. doi: 10.1136/jech-2019-212825

E-Table 11

E-Table 11: Ethnicity and Post-stroke Dementia Cox regression analysis- partially adjusted, for hes linked data

	Adjusted HR for age, sex, imd	95% CI		lrtest p-value	N
		Lower	Upper		
**Ethnicity***				0.001	38385
White	1				
South Asian	1.344	1.054	1.712		
Black	1.637	1.240	2.160		
Mixed/Other	1.114	0.809	1.534		
**Sex***				0.434	38385
Male	1				
Female	1.025	0.963	1.091		
**Index of Multiple Deprivation (1 is least deprived)***				0.127	38385
1	1				
2	1.017	0.919	1.124		
3	1.031	0.933	1.139		
4	1.071	0.969	1.184		
5	1.122	1.017	1.237		
**Statin prescription 2 yrs prior to stroke**				<0.001	38385
No	1				
Yes	0.883	0.825	0.944		
**Uncontrolled HT (Sys=140 & Dias>=90 mmHg)- 2yrs prior to stroke**				<0.001	38385
No	1				
Yes	0.817	0.731	0.913		
**Immunosuppressive prescription 2yrs prior to stroke**				0.074	38385
No	1				
Yes	0.909	0.817	1.011		
**Antiplatelets within 90 days of stroke onset**				<0.001	38385
No	1				
Yes	0.689	0.648	0.732		
**Alcohol status**				<0.001	33568
Non-drinker	1				
Current-drinker	0.790	0.717	0.872		
Ex-drinker	0.913	0.811	1.027		
**Current smoker**				0.019	37157
No	1				
Yes	1.126	1.021	1.242		

Shiekh SI, *et al. J Epidemiol Community Health* 2019; 74:114–119. doi: 10.1136/jech-2019-212825

E-Table 11

	Adjusted HR for age, sex, imd	95% CI			
		Lower	Upper	lrtest p-value	N
**MI prior to stroke**				0.884	38385
No	1				
Yes	1.008	0.902	1.127		
**Afib prior to stroke**				0.366	38385
No	1				
Yes	1.039	0.957	1.129		
**Uncontrolled diabetes prior to stroke**				0.384	38385
No	1				
Yes	1.072	0.918	1.253		
**Consultations per year in 3yrs prior to stroke**				<0.001	38385
< 10 per year	1				
10 - 20 per year	0.833	0.776	0.895		
20 - 30 per year	0.949	0.851	1.057		
30 - 50 per year	1.126	0.967	1.312		
> 50 per year	1.530	1.073	2.181		

* Adjusted with each other

Shiekh SI, *et al. J Epidemiol Community Health* 2019; 74:114–119. doi: 10.1136/jech-2019-212825

E-Table 12

E-Table 12: Ethnicity and Post-stroke Dementia multivariable Cox regression without potential mediators (lrtest p-value 0.0372)

	Full model without Uncontrolled HT and Diabetes	95% CI	
		Lower	Upper
**Ethnicity**			
White	1		
South Asian	1.299	0.999	1.690
Black	1.465	1.065	2.014
Mixed/Other	1.121	0.777	1.617
**Sex**			
Male	1		
Female	0.981	0.913	1.053
**Index of Multiple Deprivation (1 is least deprived)**			
1	1		
2	0.953	0.850	1.069
3	0.991	0.884	1.110
4	1.068	0.953	1.195
5	1.114	0.998	1.244
**Statin prescription 2 yrs prior to stroke**			
No	1		
Yes	1.009	0.933	1.090
**Immunosuppressive prescription 2yrs prior to stroke**			
No	1		
Yes	0.944	0.841	1.058
**Antiplatelets within 90 days of stroke onset**			
No	1		
Yes	0.798	0.743	0.858
**Alcohol status**			
Non-drinker	1		
Current-drinker	0.811	0.734	0.895
Ex-drinker	0.930	0.825	1.048
**Current smoker**			
No	1		
Yes	1.198	1.079	1.329
**MI prior to stroke**			
No	1		
Yes	1.083	0.958	1.225

Shiekh SI, *et al. J Epidemiol Community Health* 2019; 74:114–119. doi: 10.1136/jech-2019-212825

E-Table 12

	Full model without Uncontrolled HT and Diabetes	95% CI	
		Lower	Upper
**Afib prior to stroke**			
No	1		
Yes	1.003	0.912	1.103
**Consultations per year in 3yrs prior to stroke**			
< 10 per year	1		
10 - 20 per year	1.009	0.930	1.094
20 - 30 per year	1.138	1.011	1.281
30 - 50 per year	1.371	1.163	1.617
> 50 per year	1.871	1.300	2.695

Shiekh SI, *et al. J Epidemiol Community Health* 2019; 74:114–119. doi: 10.1136/jech-2019-212825
